# Essential Oils as Potential Natural Antioxidants, Antimicrobial, and Antifungal Agents in Active Food Packaging

**DOI:** 10.3390/antibiotics13121168

**Published:** 2024-12-03

**Authors:** Aleksandra Bibow, Wiesław Oleszek

**Affiliations:** Institute of Soil Science and Plant Cultivation, State Research Institute, Czartoryskich 8 St., 24-100 Pulawy, Poland; wieslaw.oleszek@iung.pulawy.pl

**Keywords:** essential oils, food packaging, antimicrobial activities, biological activities, edible food packing

## Abstract

In the last few years, there has been growing interest in the harmful impact of synthetic additives, the increased consumer focus on nutrition, and their unwillingness to use antibiotics and preservatives. The food industry has been driven to seek natural alternatives to synthetic antioxidants and integrate them into the production processes. Moreover, the most significant risk factor for foodborne illness is the consumption of raw or undercooked meats and milk, which may be contaminated with *Listeria* spp., *Campylobacter* spp., or *Salmonella* spp. This article presents a review of techniques for the functional properties of biopolymer particles loaded with essential oils that form a stable network to control their release, making them ideal for improving food packaging and processing. Such substances are employed in the manufacture of packaging materials and coated films and as emulsions, nanoemulsions, and coatings directly incorporated into the food matrix. It is of paramount importance to gain an understanding of the migration mechanism and potential interactions between packaging materials and foodstuffs. A more profound comprehension of the chemical constitution and biological characteristics of these extracts and their constituents would be advantageous for the identification of prospective applications in active food packaging. The findings of our study suggest the existence of certain constraints and deficiencies in the investigation of essential oils and their efficacy in food packaging. Consequently, further comprehensive research in this domain is imperative.

## 1. Introduction

Essential oils (EOs) are volatile secondary metabolites synthesised by aromatic and medicinal plants, mainly from angiosperm families such as *Apiaceae*, *Lamiaceae*, *Rutaceae*, *Myrtaceae*, *Lauraceae*, *Verbenaceae*, *Zingiberaceae*, and *Asteraceae*. They are present in various parts of the plant, e.g., the leaves, stems, flowers, seeds, fruit, bark, and roots and give the plant its characteristic smell, taste, or both [1]. EOs are typically liquid at room temperature, although some may be solid, gaseous, or resinous, and exhibit a wide range of colours, from pale yellow to emerald green, blue, dark brown, and red. The majority of EOs are obtained through the process of hydrodistillation. The principal benefit of this approach is that it enables the aromatic compounds in the fragrances to be transformed into a vapour state without undergoing decomposition. Hydrolats, also known as floral waters, are the by-product of the production process. The other most common method is steam distillation. The method entails the passage of steam through the plant material. The steam entrains and lifts the volatile molecules present in the plant material. EOs can also be produced by solvent extraction or microwave-assisted dry distillation. The essential oil is produced through distillation, while other extracts may contain volatiles but are not classified as essential oils [2]. Consequently, it is difficult to determine which method of essential oil extraction is the most appropriate for a specific purpose. The various methods of EO extraction each possess distinct advantages and disadvantages and are employed in a multitude of contexts and for a diverse range of purposes [3].

Essential oils are a good source of bioactive compounds, with antioxidant and antimicrobial properties, and are gaining increasing attention as natural additives for extending the shelf life of food products. The usage of a combination of packaging technology and the antimicrobial properties of essential oils refers to the terminology of active food packaging. Active packaging is the intentional inclusion of an active agent in the packaging system, either in the packaging material itself or in the packaging headspace. This technology allows for the release of active compounds into the packaging atmosphere and the absorption of unwanted substances from the environment, as well as the inclusion of scavengers (viruses, bacteria, fungi) in the packaging [4].

Active packaging can enhance the safety and quality of food by reducing microbiological contamination and preserving sensory properties, thus extending the shelf life of the product. Raw meat is particularly vulnerable to microbiological contamination, often by pathogens like *Listeria monocytogenes* and *Escherichia coli*, which can cause food poisoning. Cellulose paper is commonly used for packaging raw meat, but it can be enhanced into “active paper” by coating it with antimicrobial substances. When applied to the paper, plant essential oils such as cinnamon, tea tree, or clove can serve as natural preservatives. The EOs of cinnamon bark and clove, as well as the extract of clove, are capable of disrupting the cell wall and cytoplasmic membrane of bacteria. This enables the active constituents of the oils to gain access to the cells, ultimately leading to cell death. The results demonstrated that paper sheets with a coated layer of oil obtained a thickness of w100 μm and reduced the growth of *Listeria innocua* and *Escherichia coli* during cold storage. The colour of the meat remained unchanged at 4 °C for 72 h. The results indicate the potential use of essential-oil-coated paper as active packaging for raw meat [5]. Essential oils from spice plants have been extensively researched for their potential to enhance food quality and safety. EOs with antimicrobial properties are of particular interest, as they could be used as natural preservatives. It would be beneficial to gain a deeper comprehension of the chemical composition and biological characteristics of these extracts and their constituents to identify potential applications in active food packaging.

## 2. Essential Oil (EO) Sources

Some examples of plants and their major volatile compounds are shown in Table 1. Notably, certain plants produce more than one type of oil. For example, oranges contain distinct types of oil: some in the fruit’s skin, another in the leaves, and others in the flowers. A review of the published research indicates that oregano, thyme, mint, rosemary, coriander, cinnamon, rose, clove, sage, cedar, sandalwood, pine, and lavender essential oils (EOs) exhibited the highest antibacterial activity.

The composition of essential oils is highly variable, both qualitatively and quantitatively. This variability is a consequence of the complex chemical processes involved in the extraction and production of essential oils. The intrinsic factors influencing this variability are related to the plant itself. For example, essential oils extracted from seeds and immature leaves of the same plant show different chemical profiles [6]. Moreover, the interaction of the plant with the surrounding environment, including soil type and climate, as well as the maturity of the plant at the time of harvest, also affects the variability [7]. Further factors are external to the extraction method and the environment in which it is carried out. Determining the yield and composition of EO therefore depends on many factors. In some cases, it is difficult to distinguish between these elements, as they are interrelated and influence each other. All these factors have an impact on the final EO content [8]. Concerning the matter of extraction efficiency, it is estimated that approximately 200 kg of plant material is required to yield one litre of oil. To illustrate this, an example would be the production of jasmine oil, which requires handpicking around 8–10 million jasmine flowers at dawn to obtain one kilogram of essential oil. In the case of rose oil, obtaining one kg of essence requires the collection of 500 kg of petals from freshly opened flowers [9].

**Table 1:** Selected plants and their major volatile compounds.

Aromatic Species	Major Terpenes and/or Phenylpropanoids of Some Volatile Oils	References
Ageratina ligustrina (*Eupatorium semialatum* Benth.)	*δ*-elemene, farnesene, *α*-curcumene, *β*-bisabolene	[10]
Arnica (*Arnica longifolia*)	1,8-cineole, camphor, *α*-pinene, *α*-cis-bergamotene	[10]
Basil (*Ocimum basilicum* L.)	eugenol, myrcene, 1,8-cineole	[11]
Bay tree (*Laurus nobilis*)	1,8-cineole, sabinene, pinocarvone, umbellulone	[10]
Brushland shrubverbena (*Lantana achyranthifolia*)	carvacrol, *α*-bisabolol, isocaryophyllene	[10]
Caraway (*Carum carvi*)	limonene, carvone	[12]
Citronella (*Cymbopogon winterianus*)	citronellal, citronellol, geraniol and acetates	[12]
Clove (*Syzygium aromaticum*)	eugenol, *β*-caryophyllene, *α*-humulene	[10,13]
Cinnamon (*Cinnamomum verum*)	eugenol, trans-3-caren-2-ol, *α*-pinen	[10]
Cinnamon (*Cinnamomum zeylanicum*)	trans-cinnamaldehyde	[10]
Chaste tree (*Vitex agnus-castus*)	1,8-cineole, *β*-farnesene, manool	[10]
Chamomile (*Matricaria chamomilla*)	azulene, *α*-bisabolol, chamazulene, *β*-farnesene	[12]
Chilca (*Baccharis latifolia*)	limonene, sabinene, *α*-pinene, *β*-pinene	[10]
Coleus amboinicus (*Plectranthus amboinicus*)	carvacrol, ρ-cymene, thymol	[10]
Coriander (*Coriandrum sativum*)	linalol, geraniol	[10]
Cornmint (*Mentha arvensis*)	menthol, methyl acetate	[10]
Croton (*Croton cajucara* Benth.)	linalol, trans-crotonin, cis-cajucarin B, trans-cajucarin B, cajucarin A, cajucarinolide, isosacacarin, *α*-copaene and ciperene	[10]
Drumstick tree (*Moringa oleifera*)	pentacosane, hexacosane	[10]
Dysphania ambrosioides (*Chenopodium ambrosioides*)	m-cymene, myrtenol	[10]
Fennel (*Foeniculum vulgare* Mill.)	anethole, fenchone	[10]
French tarragon (*Artemisia dracunculus*)	methyl chavicol	[10]
Eucalyptus (*Eucalyptus L’Hér.*)	α-pinene, eucalyptol	[14]
Garlic (*Allium sativum* L.)	carvone, carvacol, p-cymene, eucalyptol	[15,16,17]
Geranium (*Pelargonium graveolens*)	geraniol, L-citronellol	[12,13]
Ginger (*Zingiber officinale*)	citral, gingerol, zingerone	[10]
*Haplophyllum tuberculatum* (no English name)	*α*- and *β*-phellandrene, limonene, *β*-ocimene, *β*-caryophyllene, myrcene	[10]
Hops (*Humulus lupulus* L.)	myrcene, limonene, *α*-pinene, *β*-pinene	[18]
Key lime *(Citrus × aurantiifolia*)	limonene, y-terpinene, trpinolene	[10]
Kaffir lime (*Citrus hystrix*)	limonene, citronellal, *β*-pinene	[10]
Lavender (*Lavendula officinalis*)	geraniol, linalool with esters	[12]
Lemongrass (*Cymbopogon flexuosus*)	citral, geraniol	[12]
Lemon verbrna (*Aloysia citrodora*)	citral, nerol, geraniol	[10]
Marjoram (*Origanum majorana* L.)	sabinene, *α*-terpinene, p-cymene, y-terpinene	[11,12]
Mexican Oregano (*Lippia graveolens*)	carvacrol, *α*-terpinyl acetate, m-cymene, thymol	[10]
Nigella, Black kumin (*Nigella sativa*)	p-cymene, *γ*-terpinene, thymoquinone, *β*-pinene	[10]
Orange (*Citrus sinensis*)	limonene,	[10]
Oregano (*Origanum vulgare*)	carvacol, thymol, linalool, *β*-myrcene, *β*-citronellol	[10,11,13]
Palmarosa (*Cymbopogon martinii*)	trans-geraniol, *β*-elemene	[12]
Sassafras (*Sassafras albidum*)	safrole	[10]
Scotch spearmint (*Mentha cardiaca*)	carvone, limonene, 1,8-cineole	[19,20]
Spilanthes (*Spilanthes americana*)	piperitone, piperitenone	[10]
Rose (*Rosa damascena*)	geraniol, rose oxide	[12]
Rosemary *(Rosmarinus officinalis*)	limonene, cineole, camphene	[11]
Rosewood (*Aniba rosaeodora*)	(+)-linalool (coriandrol) and levorotatory (−)-linalool (licareol)	[10]
Rubber rabbitbrush (*Chrysothamnus nauseosus*)	camphor, *α*- and *β*-pinene, lyratyl acetate	[10]
Sage (*Salvia officinalis* L.)	camphor, tujone	[12]
Sandalwood (*Santalum album*)	*α*-santalol, *β*-santalol	[10]
Tea-tree (*Melaleuca alternifolia*)	tepenen-4-ol, 1,8-cineole, terpinolene	[11,12]
Thyme (*Thymus vulgaris*)	thymol, carvacol, linalool, *α*-terpineol, 1,8-cineole	[10,11,13]
Thymbra (*Thymbra capitata* L.)	carvacrol, *γ*-terpinene, p-cymene.	[10]
Turmeric (*Curcuma longa*)	1,8-cineole, *α*, *β*, *γ*-curcumene, *β*-bisabolene	[10]
Willow gentian (*Gentiana asclepiadea*)	xanthones, linalool	[10]
White panicled aster (*Symphyotrichum lanceolatum*)	carvacrol, *α*-bisabolol	[10]
Vetiver (*Vetiveria zizanoides*)	vetiverol, sativen, *α*-humulene, *α*-bosabolol, farnsol	[12]

## 3. Chemical Characteristics of EOs

EOs are mixtures of volatile substances of different chemical natures. They are products of exo- and endogenous secretory tissues, which are characteristic of only certain families, genera, or species of plant. The components of the oils are present in the free state and as glycosidically bound substances. Multiple double bonds are typically three or more in alternating sequences [21].

EOs are a sophisticated mixture of numerous bioactive chemical constituents, including terpenes (camphor, camphene, carvacol, thymol, α-pinene, ρ-cymene, 1,8-cyneol, limonene, γ-terpinene, and terpinene-4-ol). Terpenoids and phenylpropanoids represent the most prevalent compounds. The EOs exhibit a slight solubility in water but a high solubility in alcohol, organic solvents, and solid oils [22]. Notably, monoterpenes frequently represent the predominant molecules in EOs, with a potential percentage of up to 90% of the total EOs composition. Also, sesquiterpenes and diterpenes may be present, albeit less frequently. Furthermore, the compounds under investigation are presented in the form of acids, alcohols, coumarins, lactones, aliphatic hydrocarbons, and acyclic esters. The chemical structure of EOs has been proven to influence their mode of action and antibacterial activity. The presence of the hydroxyl group in phenolic compounds, such as carvacrol and thymol, has been validated for its significance. These potent compounds, including carvacol and thymol, have been observed to disrupt the cell’s cytoplasmic membrane, driving proton and electron flow, enhancing active transport, and inducing the coagulation of cell contents [23]. Activities of some EOs are presented in Table 2.

## 4. Active Food Packing

There are four main categories of antimicrobial packaging. The first category involves incorporating antimicrobial compounds within a sachet or pad, which then soaks up meat exudates, inhibiting microbial growth [37]. The second category involves directly incorporating the antimicrobial substance into the packaging film using conventional heating methods such as co-extrusion, or non-heating methods like electrospinning and solvent casting. The third category involves coating the packaging with a matrix that acts as a carrier for an antimicrobial agent. The volatile active agent is released into the headspace of the packaging and onto the surface of the food through evaporation [25]. The fourth category uses polymers with antimicrobial properties, such as poly-L-lysine and chitosan. The mechanism of action for this category involves the interaction of charged amines of the polymer with the negative charges of the cell membrane of the microorganisms, causing the leaking of the intracellular constituents and resulting in cell death [26].

The food industry is constantly evolving, and so are the technologies used to package our favourite foods. Innovative packaging solutions are being developed to meet consumer demands for fresh, tasty, and convenient foods with extended shelf life and controlled quality. These changes are driven by the need to adapt to longer food distribution times due to market globalization and the shift in consumer lifestyles [38]. As we embrace these advancements, we are also addressing significant challenges in the food packaging industry. Consequently, this drives the development of new and improved packaging concepts to extend shelf life while maintaining and monitoring food safety and quality [39]. Packaging materials have evolved to include laminates, a cutting-edge development that seamlessly integrates materials with diverse properties to enhance the functionality of the final product [40]. The interaction between food and its packaging is paramount, particularly when the food comes into direct contact with the packaging. The migration process can affect the quality and safety of the food as well as its flavour. It means there is a transfer of chemical contaminants from contact materials (food packages) into the food [41]. One example of beneficial migration is seen in oxygen-scavenging films, which absorb oxygen, prevent microbial growth, and remove undesirable flavours [42]. The low-molecular-weight substances, such as additives and oligomers move from packaging films into foods by diffusion. Based on the mentioned aspects, active packaging systems utilise ethylene scavengers, carbon dioxide emitters, and antimicrobial and antioxidant components to enhance packaging materials [43,44]. Incorporating the active element directly into the packaging material through extrusion, lamination, or printing enhances product effectiveness and durability.

The food industry needs new suitable packaging materials like biodegradable coatings and films to safeguard food products. We can develop biodegradable bioplastics using abundant and renewable biobased polymer resources. These biopolymers must be cost-effective, renewable, and easily accessible [45]. The recent advancements in biopolymer-based coatings and films for active food packaging applications allow for the creation of thin films or thick coats [46]. Some of the most commonly utilised materials for developing resourceful packaging films include microbial polymers—polyhydroxyalkanoates (PHA) and poly lactic acid (PLA), wood-based polymers (lignin, cellulose, starch, hemicellulose), and protein-based polymers (wheat gluten, keratin, soy protein, gelatin and whey protein isolates). PLA has revolutionised single-use applications and disposable packaging, particularly in food packaging. Meanwhile, PHAs represent a crucial polymer family, being 100% bio-based and completely biodegradable. To ensure optimal protection against food-borne pathogens and efficient gas transport, state-of-the-art coatings and films are meticulously engineered [47].

Many EOs are deemed safe for direct use in food or food packaging and have been approved by the Food and Drug Administration (FDA) in 2016. The FDA has acknowledged GRAS (Generally Recognized as Safe) EOs from cinnamon, oregano, basil, cloves, and thyme. In the European Union (EU), the European Food Safety Authority (EFSA) is responsible for evaluating the safety of food ingredients, including essential oils. European regulations permit the use of some bioactive compounds such as eugenol, carvacrol, cinnamaldehyde, carvone, citral, thymol, limonene, linalool, and vanillin as flavouring agents.

The term GRAS is used in the context of food and food-related products [48]. EOs, volatile aromatic compounds derived from plants, fall under this category when used following established safety guidelines. The approval process entails a comprehensive review of scientific data, and only those substances that meet the requisite safety criteria are permitted for use in the EU market.

Plant-based antimicrobials such as EOs are gaining popularity as a possible support or supplement to synthetic preservatives. This is because they are environmentally friendly and considered safe for use. However, using EOs as an effective antibacterial in the food industry can take time due to their volatile nature, low solubility, and high instability [49]. The findings suggest that the process of EO encapsulation has resulted in the creation of numerous new formulations with novel applications. This process ensures the fragile oil’s protection and the active ingredient’s controlled release. The most commonly utilised carriers are polymer particles, liposomes, and solid lipid nanoparticles [3].

For instance, pectin, as a versatile ingredient, finds diverse applications in films, coatings, hydrogels, aerogels, and emulsions for food packaging. Notably, pectin-chitosan hydrogels, when combined with garlic and holy basil EOs, exhibit outstanding antimicrobial properties. Furthermore, these hydrogels are effective in preventing enzymatic browning and water loss, making them an ideal choice for food packaging materials [50]. Mendes et al. [51] demonstrated the beneficial effects of incorporating lemongrass (LGO) as an active component of pectin emulsion in cassava starch food film. This enhancement is attributed to the plasticising properties of EOs in this application. The results demonstrate that various citrus EOs, including, lime, lemon and sweet orange, were incorporated as an active component at 50% *w*/*w* relative to the dry weight, clearly illustrating the advantageous impact in applied food packaging [52].

## 5. Biological Activity of EOs in Active Food Packing

### 5.1. Antibacterial Activity of EOs in Food Packaging

Since the beginning of the 20th century, numerous EO compounds with pronounced antimicrobial activities have been subjected to rigorous scientific investigation. The antibacterial effectiveness of EOs is closely related to their ability to dissolve in fats. Essential oils and their active components are known to primarily target the cell wall and plasma membrane, interacting with cellular polysaccharides, fatty acids, and phospholipids [53]. Terpenoids and phenylpropanoids exert their antibacterial effects by interacting with the cell membrane. The activity is primarily attributed to the hydrophobic nature of cyclic hydrocarbons. This unique characteristic allows them to effectively interact with cell membranes and accumulate within the lipid bilayer of bacteria, effectively occupying the space between the fatty acid chains. The destabilisation of the cell membrane results in the loss of ions across the membrane, leading to a decrease in the transmembrane ionic gradient. Bacteria typically offset these effects by employing ionic pumps, preventing cell death. Nevertheless, a significant amount of energy is required for this process, which ultimately slows down bacterial growth [54]. Moreover, the significance of the hydroxyl group in EO’s phenolic compounds, such as carvacrol and thymol, has been corroborated [24]. Their mechanism of action is presumed to be analogous to that of other phenolics, which is generally regarded as the disruption of the cytoplasmic membrane, resulting in the impairment of the proton motive force (PMF), electron flow, active transport, and coagulation of cell contents [55].

Recent research has demonstrated the positive impact of adding neem EOs to polyester fabric [25,26]. The experiments confirmed that this addition enhanced the fabric’s ability to resist bacteria. Similarly, lavender EO is becoming increasingly common due to its enhanced antioxidant and antibacterial properties and high linalyl acetate (3.75%) and linalool (34.1%) content. Lavender oil is highly effective against Gram-positive bacteria, including *Staphylococcus aureus* and *Bacillus cereus*, while also showing some effectiveness against Gram-negative bacteria like *Pseudomonas aeruginosa*, *Escherichia coli*, and *Salmonella abony* [27,28]. The EOs derived from eucalyptus and oranges showed high activity against the pathogenic bacterium (*E. coli*, *L. plantarum*, *E. faecalis*, *S. aureus*) and low activity against the beneficial bacterium (*L. rhamnosus*, *B. subtilis*) [14].

Other research indicates that phenolic substances, including eucalyptol, have the potential to alter the structure of proteins, thereby impairing the integrity of existing bacterial biofilms and exerting an inhibitory effect on bacterial growth. Concurrently, α-pinene displays considerable bacteriostatic and free radical scavenging capabilities, as evidenced by reference [56]. Another study demonstrated that tea tree oil (TTO) and its primary component, terpinen-4-ol, exhibited antibacterial properties. These compounds were observed to penetrate and disrupt the lipid structure of the bacterial cell membranae and cell walls. The microdilution technique demonstrated that *S. aureus* strains exhibited inconsistent growth at levels exceeding 0.1% of terpinen-4-ol. Similarly, *S. epidermidis* would not increase at doses exceeding 0.2% terpinen-4-ol, while *S. aureus* would not thrive above 0.1% tea tree oil. [41,42,43]. In vitro studies have demonstrated that carvacrol and thymol exhibit the broadest spectrum of antimicrobial activity, attributed to their chemical structure [13,32]. Studies on carvacrol indicate that the presence and location of the hydroxyl group contribute to the antimicrobial and antioxidant activity. Carvacrol, thymol, and eugenol possess the phenolic structure of a hydroxyl group (-OH) attached to a carbon atom in the aromatic benzene ring [8,57]. Jeyakumar and Lawrence [58] demonstrated eugenol as another bioactive compound that exhibited antimicrobial activity, which can be an alternative to antibiotics against many microbial pathogens. Based on the findings, eugenol’s minimum inhibitory concentration (MIC) against selected pathogens is 0.0312–8 μg/mL and the minimum bactericidal concentration (MBC) is 0.0625–16 μg/mL. The results indicated that eugenol at MIC = 0.125 μg/mL and MBC = 0.250 μg/mL showed significant antimicrobial activity against *Escherichia coli* [33]. Dorman and Deans [13] observed that antimicrobial actions of carvacrol and thymol exhibit distinct patterns when confronted with Gram-positive and Gram-negative bacterial species. The observed effects of thymol against *Bacillus cereus*, *Staphylococcus aureus*, and *Pseudomonas aeruginosa* appear to be analogous to those of carvacrol. Moreover, thymol, carvacol and eugenol have been demonstrated to exert an antimicrobial effect against a diverse range of bacterial strains.

A significant correlation was found between the hydroxyl group (bound to a benzene ring) and the strong antimicrobial activity of carvacol. This demonstrated that compounds similar to carvacrol (e.g., menthol, cymene, thymol, and carvacrol methyl ester) exhibited antimicrobial activity. The hydroxyl group and the presence of a system of delocalised electrons were identified as crucial for the antimicrobial activity of carvacrol against food-borne pathogens. After the addition of carvacrol at concentration of 0.50 mol/mg of L-phosphatidylethanolamine, destabilization of the *Bacillus cereus* membrane and a decrease in the membrane potential was observed.

It has been demonstrated that carvacrol accumulates in the cytoplasmic membrane, resulting in an expansion of the membrane. Furthermore, again the hydroxyl group of carvacrol was crucial for its impact on membrane properties, as well as for its antimicrobial activity [24]. Ahmed et al. [34] reported that the antimicrobial efficacy of inulin and 15% cinnamon EOs (CEO) exhibited inhibition against tested microorganisms. The 20% CEO and inulin complex demonstrated enhanced antimicrobial activity versus both *S. typhimurium* (MIC = 3500 μg/mL, MBC = 4000 μg/mL) and *L. monocytogenes* (MIC = 4000 μg/mL and MBC = 4500 μg/mL). The antimicrobial efficacy demonstrated by the CEO in this study is in accordance with that previously documented for other essential oils, including oregano and clove. Nevertheless, both values (MIC and MBC) are markedly disparate from those documented in the existing literature [35]. Other researchers have found that cinnamon essential oil (CEO) displays robust antibacterial activity against foodborne pathogens and pathogenic bacteria in experimental models employing *Escherichia coli* and *Staphylococcus aureus*. The minimum inhibitory concentration (MIC) of cinnamon EO was observed to be comparable between the two bacterial strains (1.0 mg/mL), while the minimum bactericidal concentration (MBC) was 4.0 mg/mL and 2.0 mg/mL for *E. coli* and *Staphylococcus aureus*, respectively. Gas chromatography-mass spectrometry (GC–MS) analysis demonstrated that cinnamaldehyde was the predominant constituent in cinnamon EO, accounting for 92.40% of the total composition.

A substantial level of effort was dedicated to clarifying the mechanism of the anti-bacterial action of cinnamon essential oil (EO) against *Escherichia coli* and *Staphylococcus aureus*. This was accomplished by examining the alterations in cellular microstructure through the use of a scanning electron microscope, assessing cell permeability, membrane integrity, and membrane potential. The application of cinnamon EO at MBC levels resulted in the destruction of the cells [36]. Many studies reported that the small molecular weight of these compounds may permit their activity against both Gram-positive and Gram-negative bacteria. It has also been demonstrated that certain phenolic and non-phenolic compounds in EOs can interact with chemical groups in proteins and other biologically active molecules, including enzymes. Furthermore, non-phenolic compounds interact through an alternative functional group, such as the carbonyl group of cinnamaldehyde. The components of cinnamon and cloves demonstrated by EOs were found to bind proteins and inhibit the enzymatic activity of *Enterobacter aerogenes*. Other studies show that steam treatment of garlic results in EOs, a compound rich in thiosulfates. Garlic EOs reveal antimicrobial activity against diverse bacteria, fungi, parasites, and viruses with compounds rich in thiosulfates [15,16]. Martyn and Targonski [17] confirmed positive results in preserving and stabilising foods using EOs as natural antimicrobial agents. Their experiments improved the use of lemon balm and coriander oils to increase the shelf life of stored minced meat, mustard and garlic oils to control mould formation in packaged bread, and oregano and garlic oils to inhibit the growth of *Staphylococcus aureus, Salmonella enteritidis, Listeria monocytogenes, Escherichia coli* in whey protein. The portion of meat was mixed with both oils at a rate of 0.01% each. The added EOs affected the sensory characteristics of the meat and were perceptible in all cases. The sensory quality of meat stored at 0 °C was acceptable even after 2 weeks of storage. Coriander oil was found to have a more beneficial effect than lemon balm oil on the sensory quality of the meat. The addition of EOs had a marked inhibitory effect on the growth of *Enterobacteriaceae* and moulds. In the case of lactic acid bacteria and yeasts, there were no significant changes compared to the control sample. The pH of the stored meat was positively influenced by the addition of the oils [59]. EOs and natural additives enhance the performance of bioplastic films. These biobased reinforcements, e.g., plant EOs and natural additives, to bioplastic films significantly improve the oxygen barrier, antibacterial, and antifungal properties [60].

The research conducted by Ahari and Soufiani [45] demonstrated that eugenol and carvacrol, when employed as nanomaterials, exhibited antibacterial activity within the context of meat food packaging systems. In vitro studies showed that carvacrol exhibits antimicrobial activity, with MICs ranging from 256 to 512 μg/mL, against various types of bacteria (*S. aureus*, *Bacillus cereus*, *Salmonella typhi*, *E. coli, Klebsiella pneumonia*) and strains of *Pseudomonas* spp. The coating demonstrated a significant inhibitory effect on the growth of bacteria on the surfaces of packaged chicken. Such an outcome is a consequence of the potential for a longer shelf life. In another study conducted by Das et al. [61], improved eugenol nanoemulsion showed suitability for protecting stored food commodities in the range of 72.05–83.45 nm (size comparable with the zeta sizer-based analysis of particle size), which was attributed to ease of application and stability over an extended period. The present study demonstrated the encapsulation of eugenol in a nanoemulsion chitosan biopolymer (Nm-eugenol nanoparticle) scanned by field emission scanning electron microscopy (FE-SEM).

### 5.2. Antioxidant Activity of EOs in Food Packaging

It is known that high levels of oxygen can cause undesirable changes in various foods, such as lipid oxidation, colour changes, off-flavours, and loss of nutrients in food products. EOs and plant extracts are effective as natural antioxidants in food packaging. They are involved in incorporating active agents in the packaging to interact with, e.g., meat and its surroundings. This interaction helps trap pro-oxidant compounds or release antioxidant compounds, delaying degradation due to lipid oxidation [43]. Antioxidant packaging can be classified into independent antioxidant devices, like sachets and pads with oxygen scavengers, and packaging materials with active agents incorporated in the film or container walls.

Green tea extract (0.5%) was used in distiller dried grains protein films to evaluate peroxide value (POV) and thiobarbituric acid reactive substances (TBARSs) during 10 days of storage of pork meat. The study reported an increase in POV in a day for the control and 3 days for the treatment, indicating a delayed effect of tea extract inducing lipid oxidation. Results show that keeping oxygen concentration below 0.15% can significantly inhibit these deterioration processes. An application of tea extract had a delayed effect in inducing lipid oxidation [62,63]. It has been demonstrated by other authors that green tea displays a notable antioxidant efficacy as a free radical scavenger. It is a rich source of polyphenolic compounds, including catechins, theaflavins, and gallic acid. These substances are capable of scavenging free radicals present in reactive oxygen and nitrogenous species. The predominant hydrogen donor antioxidants are those with a phenolic structure. Green tea extract has been demonstrated to function as an efficient antioxidant even when it is not in direct contact with food [62]. Two pathways have been established as the means by which catechins attain their total antioxidant capacity (TAC): the donation of a hydrogen atom and the transfer of a single electron. The functional film, comprising green tea extract, is produced via an adsorption process. To assess the antioxidant potential of the film, DPPH (2,2-diphenyl-1-picrylhydrazyl) and ORAC (Oxygen Radical Absorbance Capacity) studies were undertaken utilising fresh minced beef as the test subject. The minced meat samples were packaged using the active film and stored for 23 days at 4 °C under a modified atmosphere. Moreover, the active solution exhibited a concentration of 47.62 μg/g of bioactive compounds, with no notable discrepancy detected across the samples stored for either 0 or 23 days. Furthermore, the active packaging on day 19 was observed to maintain the redness of the meat at a level comparable to that of the blank packaging on day 6. Based on these findings, it can be concluded that the active film has the potential to impede the oxidation of myoglobin [64]. Similar results were reported by Siripatrawan and Harte [65] who showed that a chitosan film incorporated with green tea extract (GTE) has the potential to be utilised as an active film. The results demonstrated that as a natural antioxidant, GTE improved antioxidant properties at concentrations of 20%.

### 5.3. Antifungal Activity of EOs in Food Packaging

EO’s variable bioefficacy gave rise to the idea of using EOs in the management of stored food. The efficacy of *Mentha cardiaca* essential oils (EOs) as a protective measure against fungal and aflatoxin B1 (AFB1) degradation in stored dry fruit was examined. There is a substantial body of research examining the use of essential oils (EOs) as a biological fungicide in laboratory and food systems models. A study was undertaken to investigate the potential of *Mentha cardiaca* essential oils (EOs) as a natural, environmentally-friendly alternative for the protection of stored dry fruits against fungal contamination. The in vitro investigation showed that the antifungal and anti-aflatoxigenic activities of *Mentha cardiaca* EOs were found to be 1.25 and 1.0 μL/mL, respectively [45]. The EOs showed promising phenolic content (7.52 μg gallic acid equivalent) and significant free radical scavenging activity. In vitro, they protected stored fruits from mould infestation and aflatoxin B1 contamination. The EOs also exhibited inhibitory effects on fungal growth at a concentration of 2.0 μL/mL, with the minimum concentration required for fungicidal action being 4.0 μL/mL [20]. The different results show that lavender EO treatment is effective against several moulds. The antifungal effect is enhanced by the predominance of linalyl acetate. After the lavender EO with a dose of 0.8 mg/cm^2^ treatment of paper, *Aspergillus brasiliensis* and *Fusarium moniliforme* remained between 40–50% at the five-day periods [29]. Other investigators have demonstrated antifungal activity against *Aspergillus niger*, *Aspergillus flavus*, *Aspergillus parasiticus*, and *Penicillium chrysogenum* using composite bioactive chitosan films infused with essential oils derived from thyme and oregano. These films have been shown to inhibit fungal growth. The bioactive films demonstrated a gradual release of volatile components (26%) over a 12-week duration at 28 °C, with no notable alteration in aroma, flavour, visual appearance, or overall perception when compared to the control sample of unsupplemented rice. The analysis of the sensory characteristics of rice samples containing bioactive chitosan-based films with embedded essential oil nanoemulsions revealed no statistically significant (*p* > 0.05) alterations in aroma, flavour, visual appearance, or overall acceptability [29].

## 6. EOs in the Edible Packing

One effective method to decrease plastic waste is to prioritise the use of alternatives, such as biodegradable packaging and edible coatings and films. Edible packages (EPs) offer a sustainable solution for reducing plastic consumption. These innovative packages, in the form of coatings or films, not only help to preserve food but also offer the opportunity to incorporate bioactive compounds that enhance both technological and biological properties [29,66]. In accordance with the European Directive (1998) [67] and FDA Rules (2024) [68], edible films and coatings are routinely classified as food additives, packaging materials, and coating ingredients. Active systems are required to comply with the regulations established by regulatory organisations, including the European Food Safety Authority (EFSA; EU), the Food and Drug Administration (FDA; USA), and with national and state-level regulatory bodies. This alignment encompasses safety, utilization, and marketing efforts. Moreover, edible packaging, including films and coatings, presents a sustainable and inventive solution for food preservation [48,69]. Based on findings, the biopolymers with generally recognised as safe (GRAS) additives and a packaging thickness of 0.3 mm or less are suitable for food products, including fruits, vegetables, grains, meat, poultry, and eggs. Embracing this technology can lead to reduced food waste and environmental impact [70].

Biopolymer films are cost-effective, biocompatible, and functional. By modifying the biopolymer matrix with antimicrobial and antioxidant oils, it is possible to use the produced films in food packaging instead of synthetic materials [71]. It is possible to incorporate EOs into the mixture, thus improving the functional and physicochemical properties [72]. The utilisation of active packaging materials has the potential to reduce the diffusion rate of EOs as a natural protection in food packing.

According to the literature, the antimicrobial effects of EOs in combination with other agents can be used in emulsions, preservation methods, films, coatings, and vapour phases as an antimicrobial agent against foodborne pathogens [73]. The utilisation of cellulose nanocrystals (CNC) as a stabiliser and amplifier in conjunction with bergamot essential oils (EOs) within whey protein isolate (WPI) films has been demonstrated to not only enhance the physical and mechanical attributes of the film, but also to augment the antibacterial efficacy of the bergamot Eos. The use of CNC as an additional component in EOs hybrid films appears to be a potential approach for enhancing the physical, mechanical, and antibacterial characteristics of EFP films. Ranjbaryan et al. [36,74] investigated the impact of CNF incorporation on the antioxidant and antimicrobial properties of SC–CEO composite films. They developed cinnamon EOs (CEO), nanoemulsion (CNF), and sodium caseinate (SC) films. The findings demonstrated that the incorporation of cellulose nanofibrils (CNF) could serve as a stabilising agent, enhancing the controlled release characteristics of carnosine (CEO) in SC–CEO composite films and augmenting the film’s antioxidant attributes. Mujtaba et al. [75] reported a significant increase in the antioxidant and antimicrobial properties of mucilage films when CNF was increased from 0 to 6%. The incorporation of CNF improved the release behaviour and solubility of the film matrix. Importantly, CNF as a controlled release agent and stabiliser is a promising filler to improve the antioxidant and antibacterial properties of edible food packaging (EFP) films. It is important to note that biobased materials are the perfect choice for creating food packaging because of their biodegradability, biocompatibility, and low toxicity. These materials offer good barrier properties and antimicrobial and antioxidant activities, making them ideal for preserving different food products. Combining polysaccharides and proteins can create composite films with improved mechanical and biological behaviour, providing a sustainable and effective solution for food packaging needs [76].

This was also observed that the films with significant content of grape seed extract and *Ziziphora clinopodioides* essential oil (ZEO) improved total phenolic content, antimicrobial and antioxidant activities, thickness, and water vapour barrier properties. Incorporating ZEO into chitosan and gelatin films has demonstrated effective antibacterial activity against Gram-positive and Gram-negative bacteria. The antibacterial effect of ZEO has been attributed to the presence of carvacrol and thymol [77]. The highest antioxidant activity was observed in the chitosan enriched with 1% ZEO in combination with 1% GSE (grape seed extract) (45.21%), which exhibited the greatest antioxidant capacity.

Similar results were found in a study with gelatin-nanochitosan films (GNC) containing *Zataria multiflora* Boiss essential oil (ZMEO). They slowed bacterial in vitro growth *(Listeria monocytogenes* and *Salmonella typhimurium)* in chicken meat. Based on findings, applying GNC films containing 0.6 or 0.9% ZMEO to chicken breast meat demonstrated antibacterial activity against *S. typhimurium*. Using GNC films containing 0.9% ZMEO resulted in an extension of the shelf life of chicken breast meat during refrigerated storage, with a minimum duration of 14 days. Some researchers have reported comparable outcomes, indicating that films and coatings comprising a variety of EOs may potentially inhibit the proliferation of (psychrotrophic bacteria) PSC in refrigerated meat products [78]. Similar results were found in a previous study in which a significant correlation was found between developed incorporated antioxidant chitosan-based edible films with *Zataria multiflora* Boiss (ZEO) and grape seed extract (GSE) in combination and alone. To illustrate, the spectrophotometric analysis revealed that the absorbance of 10 g/L GSE, 10 g/L GSE + 5 g/L ZEO and 10 g/L GSE + 10 g/L ZEO chitosan films were 1, 2 and 2 at 700 nm, respectively, and 2, respectively. The inclusion of 10 g/L ZEO in GSE film resulted in an enhancement of the water vapour transmission rate of chitosan composites. Furthermore, the incorporation of GSE and ZEO within chitosan matrices led to an increase in surface hydrophobicity, comprehensive phenolic content, and antioxidant efficacy. The findings indicated that the formulation of chitosan film with GSE could be employed as an efficacious film at medium moisture content, particularly in the context of meat products, due to its noteworthy values for the swelling ratio and antioxidant properties [79].

The research by Simona et al. [80] solidly establishes the physicochemical and biological properties of carrageenan-based films containing orange essential oil (OEO) and trehalose. These innovative films reduce UV transmittance and exhibit remarkable resistance to Gram-positive bacteria, specifically *S. aureus*. The results showed that when OEO is combined with trehalose, it reduces the transmission value in the UV and visible light range up to 0.14% ± 0.02% at 356 nm. This means that the films produced can act as protectors against UV rays. FTIR and SEM analysis demonstrated that the quantity of carrageenan utilized, as well as the combination of OEO, significantly influenced the compatibility of trehalose with the film matrix. These results suggest that trehalose was found to be dependent on the amount of carrageenan, the presence of the additional oil and the addition of Tween (non-ionic surfactant). The majority of the EFP demonstrated sensitivity to Gram-positive bacteria (*Staphylococcus aureus* subsp. *aureus*). In the study conducted by Cai et al. [81], tea tree essential oil (TTO) was loaded into gliadin (Gli) nanoparticles and incorporated into antibacterial food packaging via glycyrrhiza polysaccharide (GP) nanofibers. The aim was to inhibit the expansion of *Salmonella typhimurium* on the exterior of meat products. To stabilise Gli nanoparticles, researchers used gum arabic (Ga). Fourier infrared spectroscopy (FTIR) results demonstrated the successful encapsulation of TTO. The application of Gli/Ga-TTO@GP nanofibers inhibited the growth of *Salmonella typhimurium* on pork and chicken samples by 98.52% and 97.86%, respectively, over a five-day storage period. The results corroborated the hypothesis that Gli/Ga-TTO@GP nanofibers exerted an inhibitory effect on *Salmonella typhimurium*, and demonstrated the robustness of TTO activity.

## 7. Conclusions

The present work contributes to a better understanding of essential oils’ potential natural antioxidant, antibacterial, and antifungal activity in the active food packaging industry. A potential source of naturally occurring antimicrobials are essential plant oils. Most EOs are currently classified as generally recognised as safe (GRAS) by the US Food and Drug Administration (FDA) and are consequently permitted as food additives and preservatives. They are now perceived as a potentially valuable source of natural products for the development of antimicrobial agents in active food packaging systems. The practical application of EOs can help establish a healthy and well-maintained food environment. It would be beneficial to gain a deeper comprehension of the chemical composition and biological characteristics of these extracts and their constituents, to identify potential applications in active food packaging. A substantial opportunity for the regulation of pathogen proliferation during food storage lies in the advancement of biological activity films that exhibit augmented structural attributes and antimicrobial capabilities. However, despite their recognised safety, there are no specific regulations governing their use, and existing ordinances do not allow for standardization. It is essential to comprehend their properties, specific modes of action, and their impact on the components of the food produced as antioxidants in food production. Furthermore, it is imperative to ascertain the precise dosage and safety standards for their utilization. While underscoring their applicability, additional, more comprehensive research into their antimicrobial properties for use in food packaging is undoubtedly warranted.

## Figures and Tables

**Table 2:** Activities of EOs.

EOs Sources	Active Compounds	Activity	Organism Tested	References
Neem	carvacol, thymol	anti-bac	*Escherichia coli*, *Salmonella abony*	[24,25,26]
Lavender	geraniol, linalool, α-pinene, *β*-pinene, *β*-phellandrene, camphene linalyl acetate	anti-bac anti-fungal	*Staphylococcus aureus*, *Bacillus cereus*, *Pseudomonas aeruginosa*, *Salmonella abony*, *Escherichia coli* *Aspergillus brasiliensis*, *Fusarium moniliforme*	[27,28] [29]
Orange	limonene, myrcene, α-pinene	anti-bac	*Escherichia coli*, *Lactobacillus plantarum*, *Enterococcus faecalis*, *Staphylococcus aureus*, *actobacillus rhamnosus*, *Bacillus subtilis*	[14]
Eucalyptus	α-pinene, eucalyptol, 1,8-cineole, citronellal, citronellol,	anti-bac	*Escherichia coli*, *Lactobacillus plantarum*, *Enterococcus faecalis*, *Staphylococcus aureus*, *Lactobacillus rhamnosus*, *Bacillus subtilis*	[14]
Tea tree	terpinen-4-ol, carvacol, thymol, eugenol	anti-bac	*Staphylococcus aureus*, *Escherichia coli*, *Bacillus cereus*, *Pseudomonas aeruginosa*	[30,31,32,33]
Cinnamon	eugenol, trans-3-caren-2-ol, *α*-pinen, trans-cinnamaldehyde	anti-bac	*Salmonella typhimurium Listeria monocytogenes*, *Escherichia coli*, *Staphylococcus aureus*	[34,35,36]
Garlic	carvone, carvacol, p-cymen, eucalyptol	anti-bac	*Escherichia coli*, *Staphylococcus aureus*, *Pseudomonas aeruginosa*, *Listeria Innocua*	[15,16,17]
Thyme	thymol, carvacol, linalool, *α*-terpineol, 1,8-cineole	anti-fungal	*Aspergillus niger*, *Aspergillus flavus*, *Aspergillus parasiticus*,	[29]
Scotch spearmint	carvone, limonene, 1,8-cineole	anti-fungal	*Fusarium sp.*, *Aspergillus flavus*, *Aspergillus sydowii*	[19,20]
Oregano	carvacol, thymol, linalool	anti-fungal	*Penicillium chrysogenum*, *Aspergillus niger*, *Aspergillus flavous*,	[29]
